# Fast, sensitive method for trisaccharide biomarker detection in mucopolysaccharidosis type 1

**DOI:** 10.1038/s41598-018-22078-2

**Published:** 2018-02-27

**Authors:** Elina Makino, Helen Klodnitsky, John Leonard, James Lillie, Troy C. Lund, John Marshall, Jennifer Nietupski, Paul J. Orchard, Weston P. Miller, Clifford Phaneuf, Drew Tietz, Mariet L. Varban, Marissa Donovan, Alexey Belenki

**Affiliations:** 1 Drug Discovery, R&D, Sanofi, Waltham, MA 02451 USA; 2 Rare Disease, R&D, Sanofi, Framingham, MA 01701 USA; 3 Division of Pediatric Blood and Marrow Transplantation, University of Minnesota, Minneapolis, MN 55455 USA

## Abstract

Certain recessively inherited diseases result from an enzyme deficiency within lysosomes. In mucopolysaccharidoses (MPS), a defect in glycosaminoglycan (GAG) degradation leads to GAG accumulation followed by progressive organ and multiple system dysfunctions. Current methods of GAG analysis used to diagnose and monitor the diseases lack sensitivity and throughput. Here we report a LC-MS method with accurate metabolite mass analysis for identifying and quantifying biomarkers for MPS type I without the need for extensive sample preparation. The method revealed 225 LC-MS features that were >1000-fold enriched in urine, plasma and tissue extracts from untreated MPS I mice compared to MPS I mice treated with iduronidase to correct the disorder. Levels of several trisaccharides were elevated >10000-fold. To validate the clinical relevance of our method, we confirmed the presence of these biomarkers in urine, plasma and cerebrospinal fluid from MPS I patients and assessed changes in their levels after treatment.

## Introduction

Glycosaminoglycans (GAGs) are long, unbranched polysaccharide chains that contain N- and/or O-sulfated groups distributed along their disaccharide building blocks. Many sulfated GAG chains are covalently linked to core proteins to form proteoglycans. Located at the cell surface and in the extracellular matrix, proteoglycans perform diverse cellular functions. The majority of proteoglycans are eventually internalized and the protein portion is degraded. The GAGs are then cleaved sequentially beginning from the non-reducing end of each chain by lysosomal enzymes such as endo-β-glucuronidases or endohexosaminidases that target specific sites^1^. Endoglycosidase cleavage creates multiple terminal residues that are degraded further by unique or overlapping sets of sulfatases and exoglycosidases. A defect in any one of these enzyme activities results in the accumulation of GAGs inside lysosomes and causes one of seven types of mucopolysaccharidosis (MPS). For example, in MPS I there is a deficiency in exohydrolase α-L-iduronidase (IDUA; EC 3.2.1.76), which is required for the lysosomal degradation of heparan sulfate (HS) and dermatan sulfate (DS). MPSI is clinically subdivided into Hurler (MPS I-H), Hurler/Scheie (MPS I-H/S), and Scheie (MPS I-S, formerly MPS V) syndromes by the severity of symptom manifestation and rapidity of disease progression, although all three disorders represent a continuum of the same disease. The most severe form, MPS I-H, presents with multisystem developmental abnormalities that appear early in life and if left untreated, result in pre-teen fatality^2–5^.

Current assays in use to diagnose and monitor the disease progression primarily determine: GAG levels in urine, which usually decrease with treatment^6^, heparin cofactor II–thrombin complex in serum and cerebral spinal fluid^7,8^, the ratio of dermatan to chondroitin sulfate in urine^9^, or dipeptidyl peptidase IV in plasma^10^. However, these assays are relatively insensitive and laborious and most do not distinguish the seven different types of MPS. Lawrence *et al*.^11^ recently reported disease type-specific, non-reducing end carbohydrate biomarkers for MPS and proposed a method for detection that involved depolymerization of GAG chains with endolytic enzymes and reductive amination with isotopically tagged aniline.

In this paper we describe a faster, more sensitive method of detecting several biomarkers for MPS I that does not require GAG depolymerization and labelling. The same approach could be used for distinguishing MPS type and for diagnosing other lysosomal storage diseases. This method should therefore be extremely useful for early disease detection, including newborn screening, and for tracking disease progression and response to therapy.

## Materials and Methods

### Reagents

For murine studies, recombinant human α-L-iduronidase from Genzyme Corporation (Cambridge, MA) was used. For *in vitro* studies, recombinant human α-L-iduronidase/IDUA Protein (CF 4119-GH-010), recombinant human iduronate 2-sulfatase protein (CF 2449-SU), and recombinant mouse sulfamidase/SGSH Protein (CF 2969-SU-020) were obtained from R&D Systems.

### Cell lines

Human skin fibroblasts from Hurler, Hurler-Scheie and Scheie patients as well as from MPS II and MPS III patients (GM00798, GM02846, GM00963, GM01256B, GM01323A) were purchased from Coriell (Camden, NJ). Fibroblasts were grown in medium containing: MEM (ATCC, Catalog # 30–2003), 15% fetal bovine serum, 50 U/ml penicillin, 50 μg/ml streptomycin, 10 μg/ml piperacillin, and 10 μg/ml ciprofloxacin.

### *In vivo* experiments

Hurler mice homozygous for a nonsense mutation at codon 392 in *Idua* (W392x mutation^12^) were licensed from the University of Alabama. Heterozygous littermates were asymptomatic and served as controls. All mice were housed under pathogen-free conditions in an animal facility at Sanofi Genzyme according to IACUC approved protocols. Procedures involving mice were reviewed and approved by Sanofi Genzyme’s Institutional Animal Care and Use Committee following guidelines established by the Association for Assessment of Accreditation of Laboratory Animal Care (AAALAC).

### Enzyme Replacement Therapy (ERT) studies in mice

Hurler mice (5–6 weeks old, 3 male and 3 female/per group) were injected weekly via tail-vein with recombinant human α-L-iduronidase (Genzyme, Cambridge, MA). The enzyme was diluted with 0.9% saline and the injections were performed at doses of 0.001, 0.01, 0.1 or 1 mg/kg to provide an efficacy curve. Livers were harvested four days after the final injection and processed for GAG quantification. A second study was performed to determine the duration of the effects of ERT. Hurler mice (8–10 weeks old) received a single injection via tail-vein of recombinant human α-L-iduronidase at a dose of 0.1 mg/kg. Livers were harvested 2, 4, 8 and 16 weeks post enzyme administration and processed for GAG quantification.

### Heparan sulfate (HS) assay method 1

HILIC column methods were used for pelleted cells and samples from mouse liver, urine and mouse plasma. Biological samples were solubilized in 0.1 M sodium acetate buffer, pH 7.0, containing 0.1 mM calcium acetate, 0.1% Triton-X100 and protease inhibitors cocktail (Sigma-Aldrich, Cat # P8340) (Buffer A). After 10 minutes of incubation on ice, cell lysates were sonicated (Q Sonica, Newtown, CT: amplitude 75, process time 4:00, pulse-ON Time 00:20, pulse-OFF Time 00:10) and cleared by centrifugation. The supernatant was digested with heparinases (I, II, and III, catalog # H2519, H6512, H8891, respectively, Sigma-Aldridge) for 72 hours at 37 °C with agitation. The digests were filtered through 10 kDa cut off filters (PALL life Sciences, Centrifugal Devices, Nanosep 10k omega, OD010C34) and analyzed by LC-MS. Specific disaccharides derived from HS were analyzed using an Applied Biosystem API-5000 MS/MS System interfaced to a Waters Acquity Classic UPLC. Chromatography was performed using a Phenomenex Luna 3 u HILIC 200 A, 100 × 4.6 mm column. Mobile phase A (MPA) consisted of 90% acetonitrile, 10% water, 5 mM ammonium formate, whereas Mobile phase B (MPB) consisted of 5 mM ammonium formate in water. The column was equilibrated with 95% MPA/5% MPB followed by a gradient separation step for 8 minutes, which reduced MPA to 88% and increased MPB to 12%. Flow rate was 1.0 ml/min and column temperature was 15 °C. Eight major disaccharide products of heparan sulfate digestion were quantitated using standards purchased from Dextra, UK (dp1-dp8). Mass spectrometry was done in negative ionization mode monitoring the following MS/MS transitions: H1001–287.5/247.6, H1002–496.2/416.0, H1003–496.3/416.0, H1004–416/137.8, H1005–538.1/458, H1006–458.4/174.9, H1007–458.4/157, H1008–378/175. The electrospray voltage was −4500 V and the source temperature was 450 °C. The concentration for each of the 8 digested disaccharides in analyzed samples was determined using their respective standard curves. The total HS value represents the sum of all 8 disaccharide amounts.

### Heparan sulfate (HS) assay method 2

A second, complementary method for HS analysis of human plasma samples consisted of ion pairing reverse phase liquid chromatography using a Waters BEH C18 150 × 2.1 mm column with 1.7 μm particle size. For the chromatographic separation, 100% MPA flowed for the first 4 min followed by a linear gradient up to 15% B over the next 4 min. The buffer composition was then changed to 50% MPA and 50% MPB and held for 2 min before returning to 100% MPA. The column was re-equilibrated with 100% MPA for 5 min before the next injection. The flow rate was 0.5 ml/min for the entirety of the method and the column was held at 65 °C. The MS/MS transitions were as follows: H1001, H1002, and H1003–496.2/416.0; H1004–416.0/137.8; H1005–538.1/458.0; H1006–458.4/282.0; H1007–458.4/327.0; H1008–378.0/175.0. The electrospray voltage was −4500 V, and the source temperature was 450 °C.

### Biomarker discovery methods in W392X murine tissues or cell culture

Samples were homogenized and solubilized in buffer containing 15 mM hexylamine (HXA) and 100 mM hexafluoroisopropanol (HFIP). Mice heterozygous for the W392x mutation were asymptomatic (Wang *et al*., 2009) and served as controls. After incubation on ice for 10 min, samples were sonicated and the supernatant was collected after centrifugation (1000 × g). An untargeted approach on a Thermo Scientific Q-Exactive Orbitrap mass spectrometer interfaced to a Waters Acquity I class UPLC was used for the analysis of liver homogenates. Ion pairing reverse phase liquid chromatography was performed using a Waters BEH C18 100 × 2.1 mm column with 1.7 μm particle size. Here, MPA was aqueous with 15 mM HXA and 100 mM HFIP. MPB consisted of a 75% acetonitrile/25% water containing 15 mM HXA and 100 mM HFIP. The analytical column was equilibrated with MPA for 0.5 min, followed by a gradient for 7 min with increasing MPB to a final MPB concentration of 50%. After each run, the column was re-equilibrated with 100% MPA for 2.8 min. Flow rate was 0.5 ml/min at room temperature. Full scan (370–1500 Da) in negative ion mode at resolving power 140 K was done for MS analysis. SIEVE software (version 1.3, Thermo Scientific) was used for downstream data processing and Spotfire (TIBCO Software Inc, Palo Alto, CA) was used for statistical analysis. The threshold value for detection of 100 instrument arbitrary units was entered for peak area for those analytes falling below the level of detection in samples from the treated condition. In such cases, the ratio of the peak area from untreated tissue sample to that of treated tissue sample would yield a lower limit. The work was done in triplicate: 3 runs for samples from treated mice, 3 runs for samples from untreated mice.

### Purification of BM652 from Hurler mouse kidney lysates

Ion pairing reverse phase liquid chromatography was performed using a Waters BEH C18 150 × 2.1 mm column with 1.7 μm particle size. MPA was aqueous with 15 mM HXA and 100 mM HFIP. MPB consisted of 75% acetonitrile containing 15 mM HXA and 100 mM HFIP. The gradient started at 100% MPA. The percentage of MPB was increased to 6% over 2 min followed by another 1 min step increase to a final concentration of MPB 20%. At the next chromatography step the concentration of MPB was increased to 75% over 1 min which concluded 1 separation run for 1 sample. The gradient was then returned to the starting conditions (100% MPA) and the column was re-equilibrated for 5 minutes before the next injection. Analyte 652 was quantified using corona charge aerosol detection^13^. Purified analyte was added to control plasma samples to create a calibration curve.

### Determination of sulfate position on the BM652

MPS I mouse liver homogenates (described in the biomarker discovery section) were used in this study. Ion pairing reverse phase liquid chromatography was performed using a Waters BEH C18 100 × 2.1 mm column with 1.7 μm particle size. Here, MPA was aqueous with 15 mM HXA and 100 mM HFIP. MPB consisted of a 75% acetonitrile/25% water containing 15 mM HXA and 100 mM. Column temperature was held at 60 °C throughout each run and injection size was 10 uL. The chromatography was run at 0.5 ml/min flow rate with the gradient starting at 100% MPA and changing linearly to 25% MPA, 75% MPB over 4 minutes. The 75% MPB condition was held for 2 minutes before returning to 100% MPA. The column was re-equilibrated for 4 minutes between injections. Thermo Scientific Q Exactive Hybrid Quadrupole-Orbitrap mass spectrometer was operated in negative mode and configured for parallel reaction monitoring with an isolation window of 2 m/z centered on 476. The HCD cell was used for fragmentation with the collision energy set to 20 eV.

### Clinical samples preparation

On the day of analysis urine, blood and CSF samples were thawed at room temperature followed by vortex homogenization. Each sample was diluted 1:10 vol:vol with MPA (15 mM HXA and 100 mM HFIP) containing 20 ng/ml of H1005 (Dextra, UK). The samples were mixed thoroughly and filtered through 10 kDa cutoff centrifugal filters at 15,000 rpm for 25 min. This study was approved by the Committee on the Use of Human Subjects in Research at the University of Minnesota, and all experiments were performed in accordance with relevant guidelines and regulations by the Committees on the Use of Human Subjects in Research at the University of Minnesota. Informed written consent was obtained for all patient samples from the parents or guardians on behalf of the child participants.

### BM652 analysis

A Waters Acquity Classic UPLC interfaced with an ABSciex API-4000 triple quad mass spectrometer was used. The analytical column was equilibrated with MPA (15 mM HXA and 100 mM HFIP) for 0.2 min at 99% MPA and 1% MPB (75% acetonitrile containing 15 mM HXA and 100 mM HFIP), followed by a gradient for 0.5 min with an increasing concentration of MPB up to 50% final. After each run the column was re-equilibrated with 99% MPA and 1% MPB, for 0.4 min. Flow was 0.7 ml/min and column temperature was 45 °C. For mass spectrometry we used negative ionization mode with the following parameters: MS/MS transition: 652.3/572.1; CE = −34.00 V; DP = −64.0 V; EP = −10.0 V and CXP = −12.0 V; voltage was −4500 V and temperature was 550 °C.

## Results and Discussion

**Figure 1:**
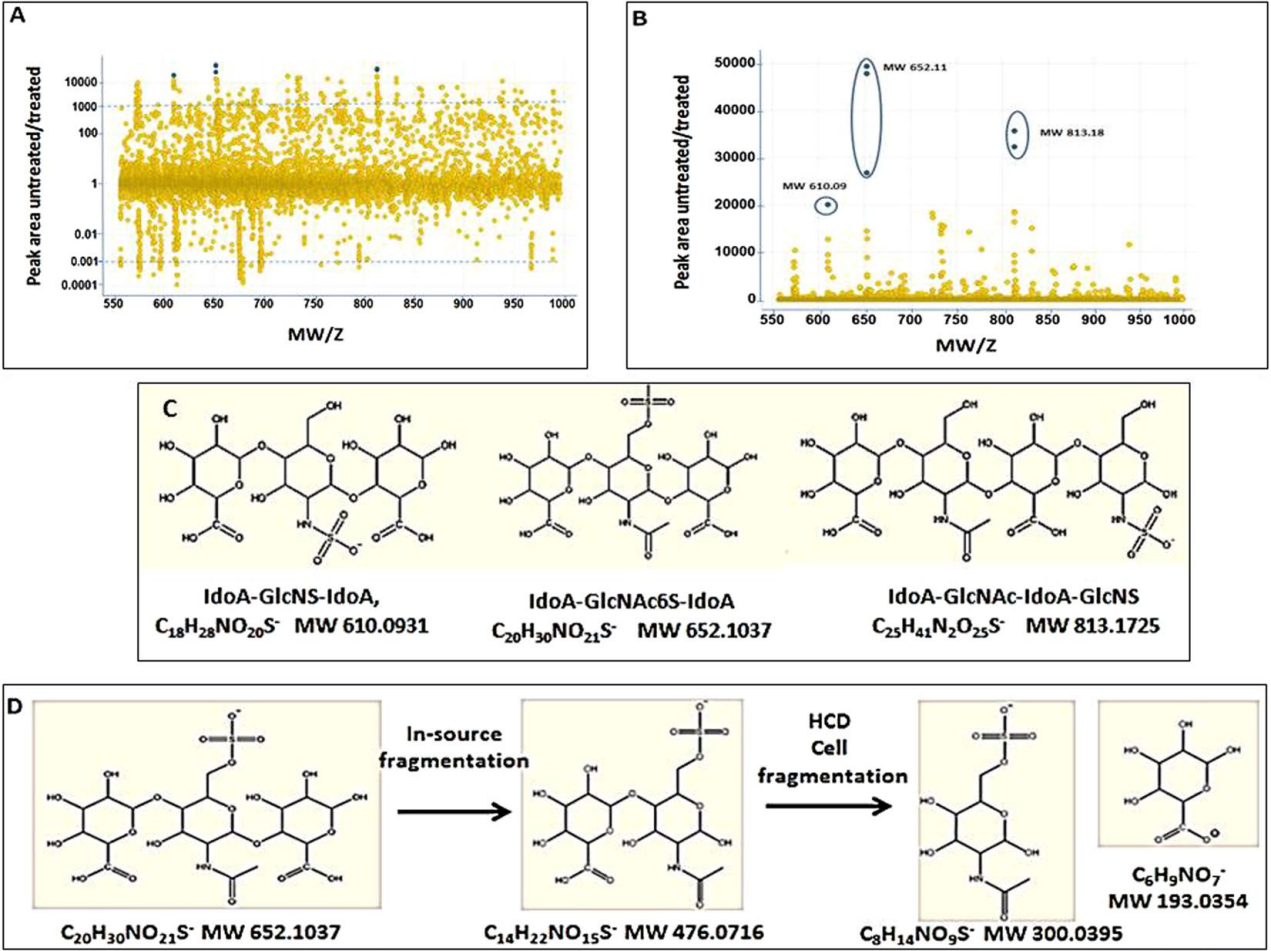
Identification of prospective biomarkers. (**A**) Spotfire analysis of 25777 features differing in molecular mass and elution time plotted as a function of abundance (ratio of peak areas) in the samples from untreated Hurler mice relative to those in Hurler mice treated with 1 mg/kg body weight of α-L-iduronidase. Dotted lines indicate ratios of peak areas that were increased or decreased by 1000-fold. 225 analytes were enriched by more than 1000-fold, 6 of which, highlighted in blue, were enriched by more than 20,000-fold. There were 90 analytes at the bottom whose levels were diminished by more than 1000-fold. (**B**) Results from (**A**) replotted on a linear scale. Three of the six highly enriched analytes had a MW of 652, two had a MW of 813 and one had a MW of 610. (**C**) Proposed structures for the 610, 652, and 813 MW analytes: IdoA-GlcNS-IdoA, IdoA-GlcNAc6s-IdoA, IdoA-GlcNAc-IdoA-GlcNS, respectively. (**D**) Fragmentation pathway of 652 analyte.

We sought a rapid and sensitive method for detecting and identifying molecules that were specifically elevated in MPS tissues and that were reduced with therapy. Previous studies^14,15^ indicated the accumulation in urine, blood and cells of MPS patient of small 550–900 Da saccharide oligomers containing 3–5 sugar building blocks. Anticipating that there would be multiple forms of sulfated oligo-sugars, differing by the number and positions of hexose and sulfate, aqueous, low molecular weight compounds were separated from homogenates of diseased tissue by liquid chromatography and analyzed by mass spectrometry. The activities of the enzymes responsible for GAG degradation are especially high in liver, so samples were drawn from mutant, Hurler (MPS I) mouse livers. MPS I is treatable by enzyme replacement therapy (ERT); cells in culture will take up endogenous α-L-iduronidase and target the enzyme to the lysosome. Cells in the body will also take up the enzyme upon intravenous administration. We therefore compared the results from Hurler mouse to those from Hurler mice that had been treated with α-L-iduronidase, rather than those from WT mice, in order to identify analytes whose levels would be sensitive to enzyme replacement therapy (ERT). 25777 features were distinguished by molecular mass and charge and elution time (Fig. 1A,B). Calculating peak areas of features with identical mass and elution times led to the discovery of 225 analytes that were more than 1000-fold enriched in extracts from untreated mice over those from treated mice. Thirtyfive of these analytes were enriched by more than 10,000-fold. None of the 225 analytes were detected in WT samples. Molecules differing in molecular weight by ~176 or 80 Da may represent glycosaminoglycans that vary in their glucuronic acid or sulfate content, respectively. Features with the same mass to charge ratio (M/Z), but differing in elution time may represent naturally occurring structural isomers such as epimers or sulfonation isomers, or may have arisen from in-source fragmentation of the parent compound. In addition, the software algorithm used for chromatographic analysis sometimes generated spurious assignments due to noise or irregularities in the shapes of the elution profiles.

Notably, there were also 90 analytes that were more than 1000- fold lower in untreated compared to enzyme-treated Hurler (Fig. 1A). Presumably, most of these analytes were normal GAG breakdown products.

Based on a correlation of their masses to those of GAG structures^11^ generated by enzymatic cleavage at known sites^16^ and the assumption that the molecules contained iduronic acid at the non-reducing end of the carbohydrate biomarker, we propose the following structures for analytes of MW 610, 652 and 813: IdoA-GlcNS-IdoA, IdoA-GlcNAc6S-IdoA, IdoA-GlcNAc-IdoA-GlcNS (Fig. 1C). These structures are consistent with the di-, tri- and tetra- saccharide products identified by different methods in earlier publications^14,15^ as biomarkers for MPS I. The primary ambiguity with the molecular structure proposed for BM652 is the location of the sulfate. Building on the assumption that the iduronic acid at the non-reducing end of our biomarker has already been processed by iduronate sulfatase, the sulfate on BM652 could either be located at the N-acetyl-glucosamine 6S position or at the 2S position of the reducing end iduronic acid. To allay the uncertainty we pursued an MS/MS strategy of detecting sulfated monosaccharide fragments of BM652. Observation of sulfated iduronic acid or sulfated N-acetyl-glucosamine, which are easily distinguishable by mass spectral methods, would reveal the location of the sulfate group.

Achieving this observation was not as straightforward as expected because fragmentation of the parent ion in the HCD cell resulted in the loss of sulfate before any glyosidic bond cleavage, thereby removing all sulfate regio-chemistry information before ion detection. However, a small amount of the BM652 parent ion underwent a loss of iduronic acid by way of in-source fragmentation to yield a disaccharide that was detected at an m/z of 476.0716. Using this phenomenon to our advantage we isolated the 476 m/z ion using quadrupole precursor ion selection, fragmented the ion in the HCD cell, and identified the monosaccharide fragmentation products (Fig. 1D). The result was the clear. There was a fragment with an m/z of 300.0395 corresponding to sulfated N-acetyl-glucosamine and no observation of sulfated iduronic acid. This result confirms that BM652 must be sulfated on the N-acetyl-glucosamine and not on the iduronic acid (Fig. 1D).

**Figure 2:**
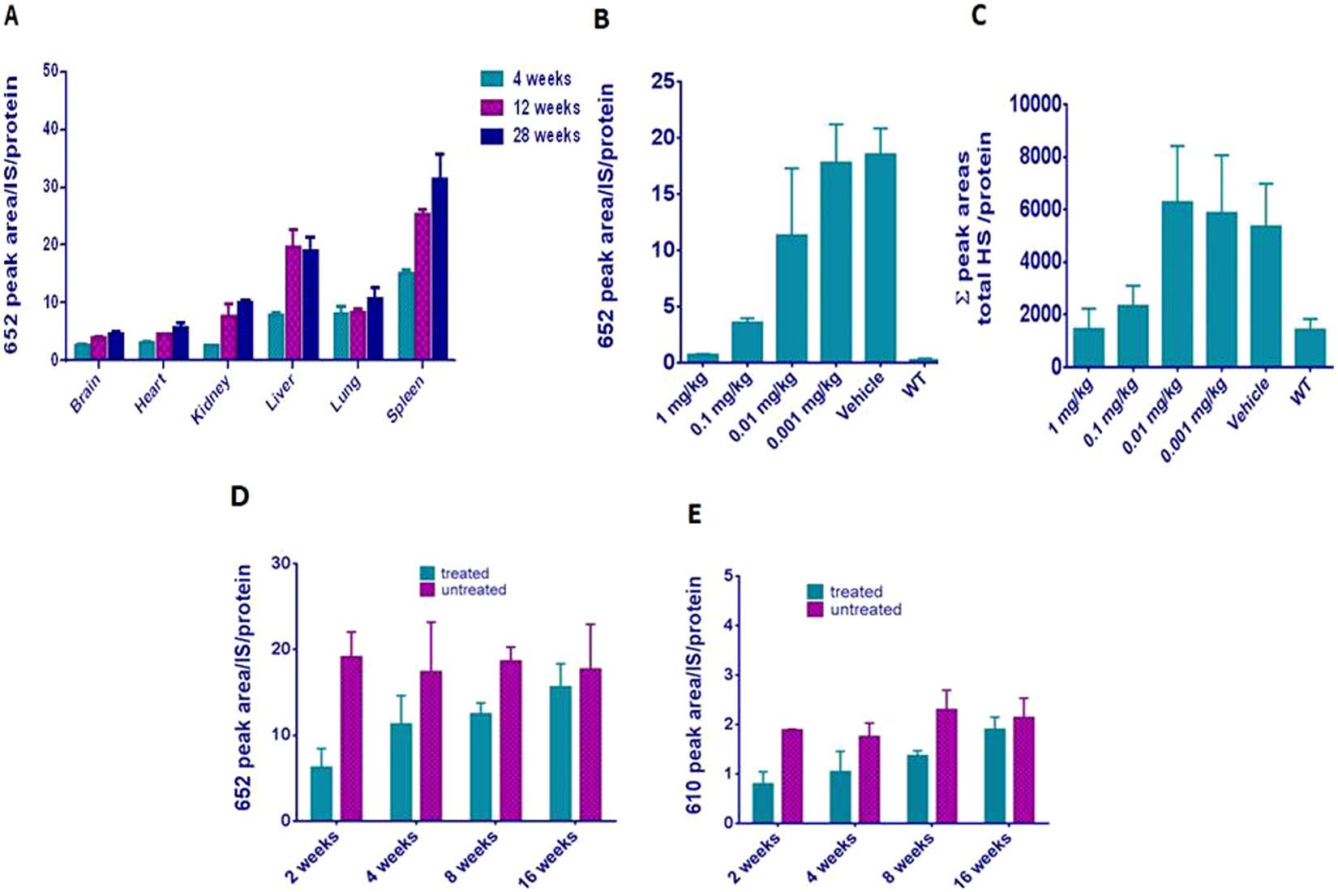
Validation of methodology in Hurler mice. Error bars show S.D. (**A**) Developmental time courses of biomarker 652 in tissues of Hurler mice. Homogenates of tissues were prepared from each of 4 mice (2 male and 2 female) and analyzed individually. (**B**) Effect of ERT treatment in biomarker 652 on total GAG levels in livers of Hurler mice. A dose range of α-L-iduronidase (0, 0.001, 0.01, 0.1, 1 mg/kg) was administered to cohorts of 6 Hurler mice (3 male and 3 female, 5–6 weeks old) via a single tail-vein injection. Four days post-administration the liver was analyzed for the 652 MW biomarker, as above. (**C**) Analysis of heparan sulfate after ERT. Levels of heparan sulfate in livers of Hurler mice showed a pattern similar to that for biomarker 652. (**D**,**E**) Temporary fall in biomarker 652 and 610 levels after ERT. Hurler mice (n = 4/time point) received a single dose of 0.1 mg/kg α-L-iduronidase at 8–10 weeks of age. Liver GAGs were analyzed at 2, 4, 8 and 16 weeks post treatment. Data are presented as mean peak area over peak area of internal standard ± S.D. H1005 (Dextra, UK) was used as the internal standard.

Levels of the 610 and 652 MW analytes were surveyed in the brain, heart, kidney, lung and spleen of Hurler mice of varying ages. Results for the sum of all 652 MW analytes are shown in Fig. 2A. Abnormal accumulation was present in all tissues, but was greater in liver, lung and spleen than in brain, heart and kidney at 4 weeks of age. Levels rose with age in all tissues, roughly tripling in kidney and doubling in liver and spleen after 12 weeks. Levels remained elevated at 28 weeks. The 610 and 652 MW analytes were present in plasma and urine (data not shown) and both were extremely low or undetectable in WT tissues at any age (e.g. in liver of mice at 4 weeks of age, Fig. 2B).Thus these two analytes, 610 MW and 652 MW, emerged as viable biomarkers for MPS I and will hereafter be referred to as BM610 and BM652, respectively.

The elevated biomarker levels in liver and spleen at 4 weeks and the dramatic increases in biomarker levels in these tissues with age mark the biomarker level in these tissues as particularly sensitive indexes of disease. To assess ERT efficacy, Hurler mice at 4 weeks of age were subjected to a single ERT treatment and tested 4 days later using our methods for analyzing BM610 and BM652. The dose-response relation for BM652 is shown in Fig. 2B. Biomarker levels were reduced in liver by more than 10-fold at a dose of 1 mg/kg and the enzyme ED_50_ was between 0.1 and 0.01 mg/kg. Comparison of BM652 measurements to those of a gold standard, heparan sulfate, showed that the changes in former replicated the response of the latter (Fig. 2C). Our methodology could also follow the useful duration of a single ERT treatment. After a single dose of 0.1 mg/kg recombinant human α-L-iduronidase, BM652 levels returned to their pretreatment levels after 16 weeks, rising to half the pretreatment level between 2 and 4 weeks post-injection (Fig. 2D). Biomarker levels remained constant in untreated mice over this period.

Measurements of the 610 MW analyte yielded results that paralleled those obtained with the 652 MW analyte: undetectable levels in control mice, elevated levels in tissues and plasma and urine (data not shown) of Hurler mice, and transiently decreased levels after a single dose of ERT (Fig. 2E). Thus levels of either analyte may be diagnostic for MPS I and show promise in establishing therapy dosage and regimen as well as in evaluating the overall benefit of the therapy.

**Figure 3:**
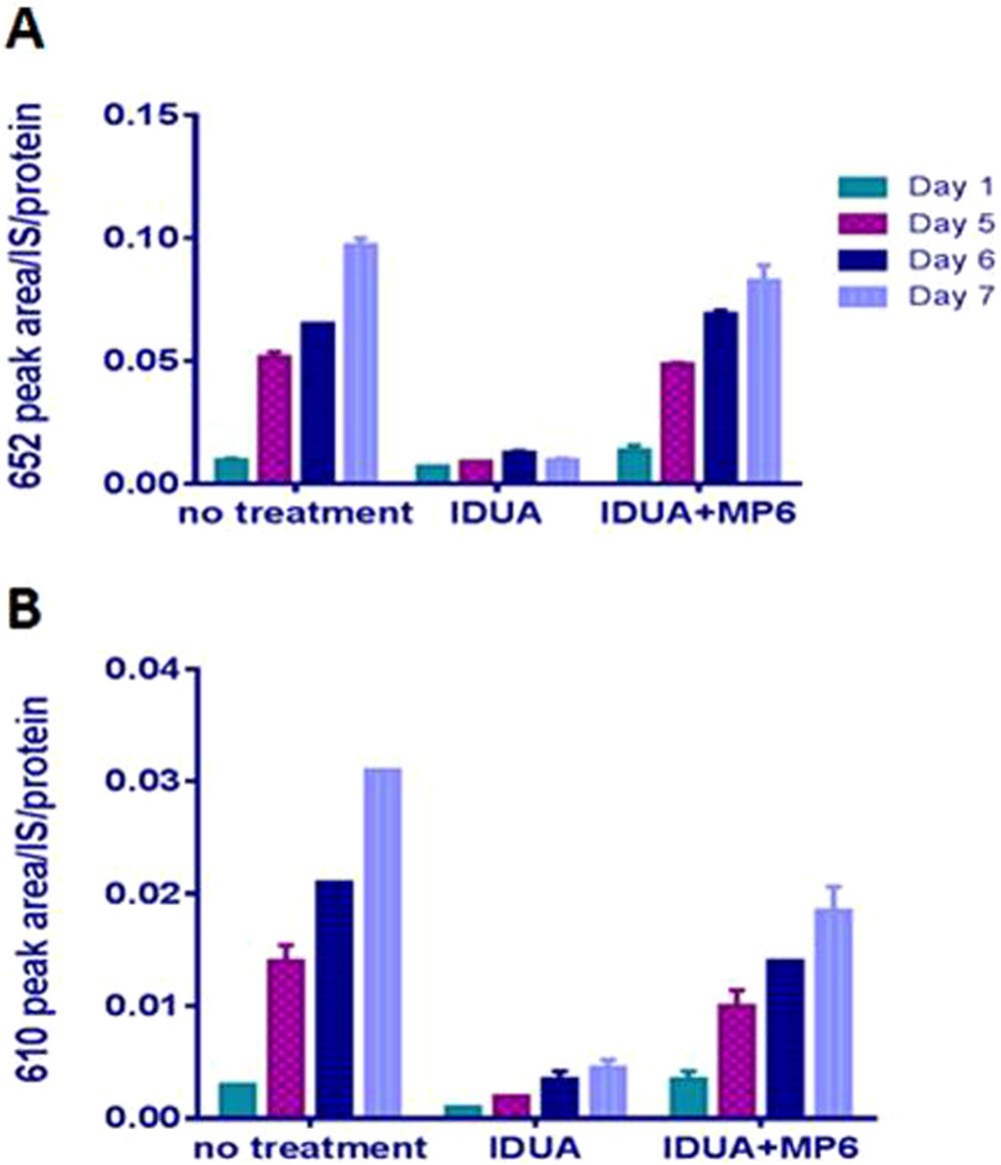
Lowered biomarker levels in human MPS I fibroblasts cultured with iduronidase. Hurler patient fibroblasts (GM00798) were treated with 0.3 µg/mL iduronidase (IDUA) for 7 days and cell media were collected 1, 5, 6 and 7 days after treatment followed by analysis for the presence of BM652 (**A**) and BM610 (**B**). As a control, 5 mM mannose 6-phosphate (MP6) was added to interfere with the entry of enzyme into the fibroblasts^27^.

We next tested whether BM610 and BM652 were similarly enriched in tissues of human patients with MPS I. Fibroblasts obtained from biopsy of a Hurler MPS I patient (GM00798) were cultured for 7 days, during which time the levels of biomarkers 652 and 610 increased approximately 10-fold (Fig. 3). BM610 and BM652 were not detected in fibroblasts of MPS IIIA or MPS V (data not shown). As expected for MPS I fibroblasts, the presence of iduronidase in the culture medium lowered biomarker contents and attenuated their rise in level over time. However, the presence of 5 mM mannose 6-phosphate to suppress receptor-mediated, cellular uptake of the enzyme^17,18^ largely blunted the response to iduronidase.

In contrast to the temporary effect of a single round of ERT, hematopoietic cell transplantation (HCT) sometimes permanently restores the defective enzyme activity. Furthermore, HCT can treat tissues not accessible to ERT, but outcomes vary^19^. A second HCT imposes significant risk, so post-transplant enzyme supplementation may represent a safer alternative. Four MPSI-H patients <14 years of age who had received hematopoietic cell transplant at least two years previously were studied. All patients were greater than 10% donor-engrafted, based on a determination of CD15 chimerism. Patients received aldurazyme at 0.58 mg/kg intravenously once a week for 24 months. The clinical endpoints and the main outcomes of this study will be reported elsewhere. Here we focus on the results of biomarker measurements.

**Figure 4:**
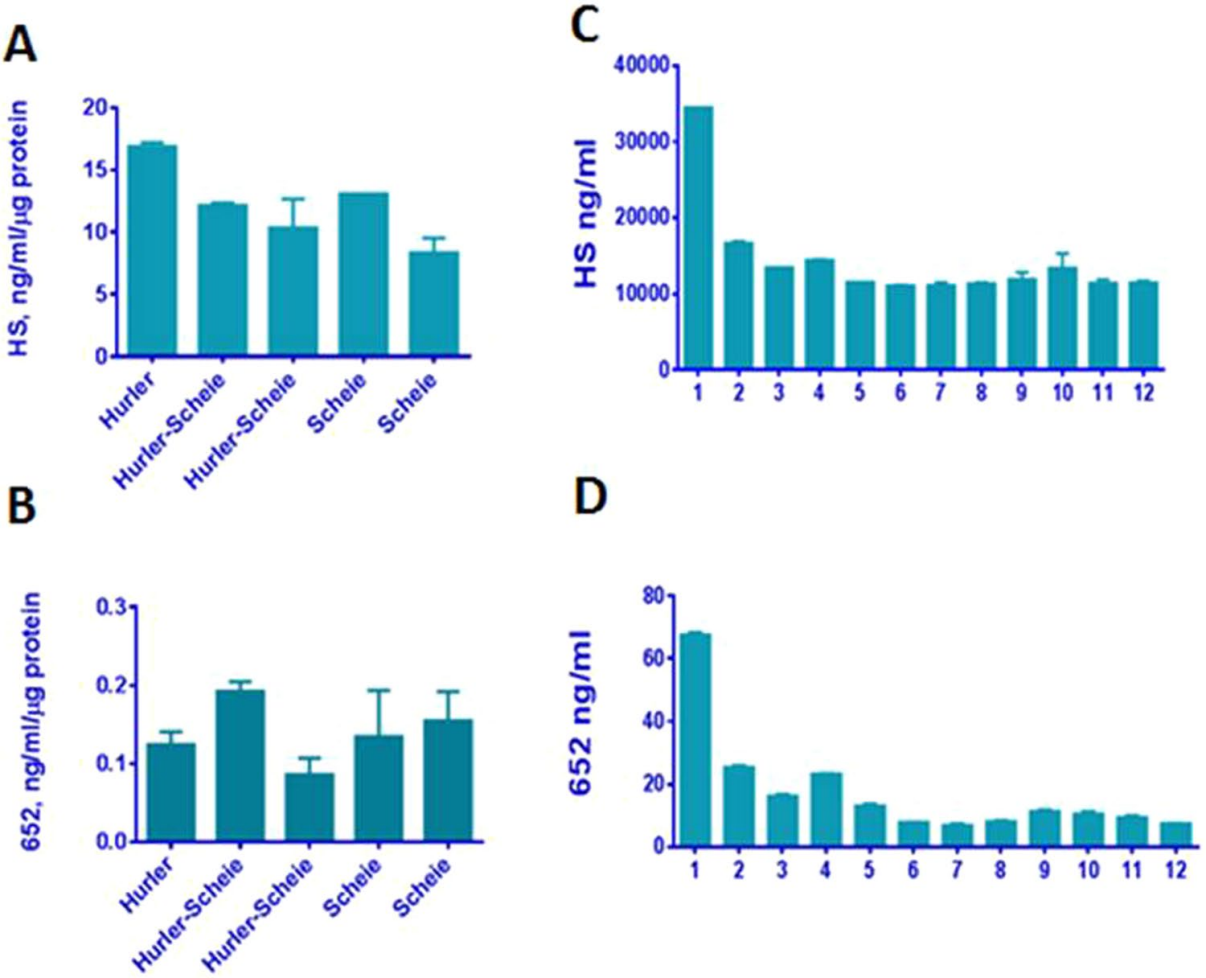
Validation of the methodology in (**A**) plasma and (**B**) urine of 4 human MPS I patients. All MPS I patients were treated >2 years after hematopoietic cell transplant (HCT) and were not receiving enzyme replacement therapy (ERT) in the interim. Patients had plasma and urine drawn at baseline (sample 1). Subsequently, patients went on ERT and had plasma and urine drawn at 3 month intervals for 24 months (samples 2 through 9). Plasma sample 6 of patient 4 is missing. n = 4 determinations were made for each sample point. (**C**) Surges in antibody titer for patients 3 and 4 as a function of time after initiation of ERT. Antibody was undetectable for patients 1 and 2 over the 24 month interval.

For practical reasons, it would be preferable to assay plasma and/or urine samples rather than biopsied tissue. GAG levels are known to be elevated in plasma and urine with MPS^20,21^ so levels of biomarkers 610 and 652 were measured. The concentrations of biomarkers 610 and 652 were so high in plasma and in urine that sample volumes of 10 µl were more than adequate for analysis. Blood and urine samples were collected every three months for a period of two years. Results for BM652 are shown in Fig. 4A and B. As was the case in Hurler mice, the overall trends were similar in plasma and urine of MPS I patients, although absolute levels were greatly elevated in urine. In patients 1 and 2, ERT following HCT lowered biomarker levels by 2- to 3-fold. However, in patients 3 and 4, biomarker levels rose to very high levels over time. Note that the undesirable outcome in 2 of 4 patients is not indicative of the success rate for ERT; patients 3 and 4 were selected specifically for this analysis because their biomarker levels did not decrease in response to therapy. The poor response could have resulted from inadequate uptake of the exogenous enzyme in all tissues and/or from the mounting of an immune response that blocked activity of the exogenous enzyme. To test for involvement of the latter, blood samples were screened for antibodies raised against iduronidase. Antibodies against iduronidase were not detected in patients 1 and 2, whose biomarker level dropped in response to therapy while iduronidase antibody titers soared in patients 3 and 4 by the time that the first samples were drawn. These results are consistent with the hypothesis that non-responding patients did indeed mount a powerful immune response within the first three months and that this response rendered ERT ineffective (Fig. 4C). Although antibody titers subsided somewhat after 12 months, biomarker levels remained high and in patient 4 climbed even higher at 24 months. The patients experienced no known clinical side effects from the rising antibodies, but these data illustrate the potential usefulness of the biomarker. The method was validated as a means for identifying MPS I patients and for evaluating the efficacy of post-HCT ERT. In the future, lack of response or rebound of biomarker levels may provide an early indication that steps need to be taken to control an immune reaction.

**Figure 5:**
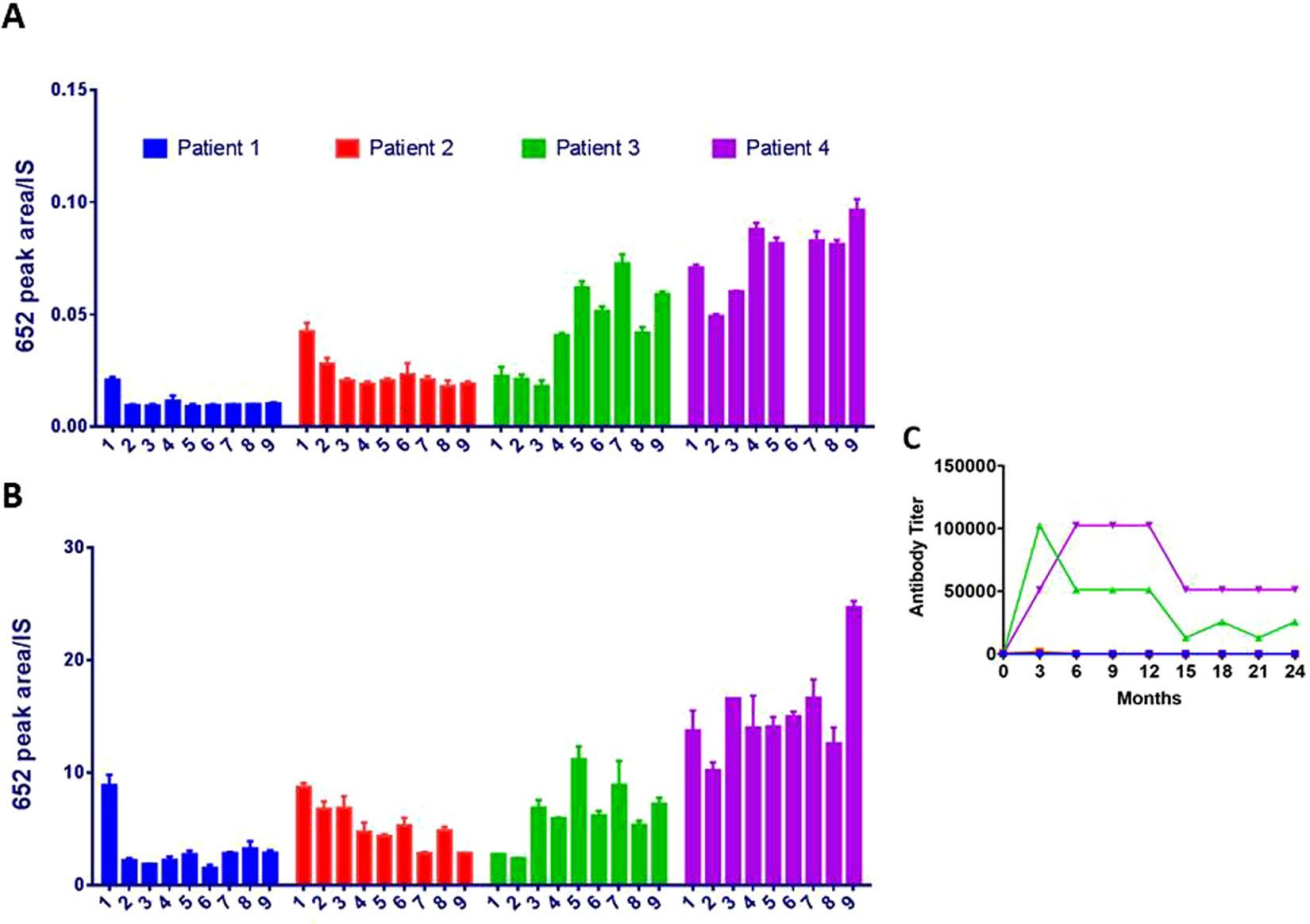
Quantification of heparan sulfate (**A**,**C**) and BM652 levels (**B**,**D**) in MPS I fibroblast cell lines (**A**,**B**) and in plasma of a human MPS I patient (**C**,**D**). Fibroblasts were derived from Hurler, Hurler-Scheie and Scheie patients. Samples 1–12 for MPS I patient (**C)** for HS and D for BM652) respectively: 1, enzyme naïve patient, 3 month prior to HCT; 2, 3 weeks pre HCT, patient on ERT; 3, 3 days prior HCT; 4, 7 days post HCT; 5, 21 days post HCT; 6, 41 days post HCT; 7, 49 days post HCT;8, 63 days post HCT; 9, 83 days post HCT; 10, 100 days post HCT; 11, 180 days post HCT, 12, 1 year post HCT.

Thus far, we have described how the biomarkers were identified and showed that their levels change in response to therapy. In order to quantify biomarker levels on an absolute scale, purified sources were needed. Ambiguity in the exact identity of the biomarker and chemical synthesis of the markers were problematic. To circumvent both issues, we purified BM652 from homogenized kidney tissues of Hurler mice (described in Materials and Methods) and used it to quantitate the amount of BM652 in MPS I fibroblasts and patient plasma samples and compared the results to total HS measurements. For *in vitro* work we tried several cell lines from patients with different disease classification (Hurler, Hurler-Scheie and Scheie). HS levels ratios may have been higher in Hurler, but a distinct pattern of Hurler > Hurler-Scheie > Scheie was not apparent for either HS or for BM652 in our small sample set of cultured fibroblast lines (Fig. 5A,B). While our method may be capable of differentiating the different subtypes of MPS I, it will require a systematic study of additional biomarkers. To that end, further work should be done to define more complete biomarker profiles for each MPS type and for each MPS I subtype.

We next quantified HS and BM652 in human plasma of an MPS I Hurler subtype patient after ERT and then as a function of time after hematopoietic cell transplantation (HCT). The levels of HS were considerably higher than those of BM652, but the trends were similar (Fig. 5C,D). Levels of both sets of molecules were 2-fold lower after ERT. Whereas HS levels were 3-fold lower after HCT and remained stable for at least a year, levels of BM652 were 4-fold lower immediately after HCT and continued to drop over time to 9-fold lower one year after HCT. The more powerful effect of HCT on BM652 than on HS may be indicative of other pathological features of the disease.

**Figure 6:**
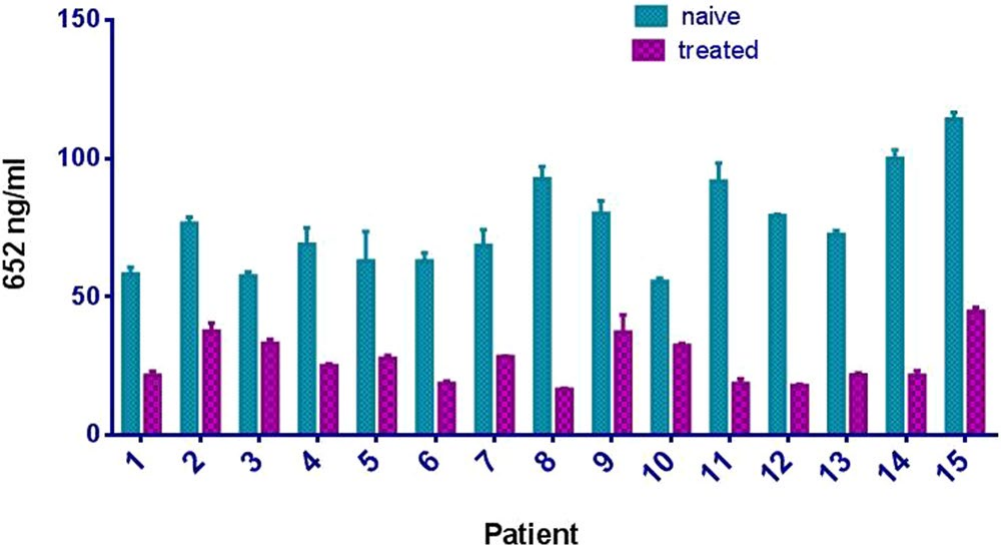
Comparison 15 patients samples, naïve and 8–10 weeks treated with ERT (IV and intrathecally). Prior to treatment, mean BM652 value was 76 ± 3; after treatment, levels dropped to 44 ± 4. On average, the decrease in BM652 level with ERT was 3.1 ± 0.3 (mean ± S.D., n = 15).

The methodology was extended to a larger group of 15 MPS I patients before and after ERT (Fig. 6). Plasma BM652 levels were high in all patients, varying over a two-fold range, from ~50 ng/ml to ~110 ng/ml. Every patient was responsive to 8–10 weeks of ERT although the improvement in biomarker level was variable; BM652 levels dropped by as little as 1.7-fold in one patient and by as much as 5.5-fold in another. On average, the mean decrease was about threefold.

**Figure 7:**
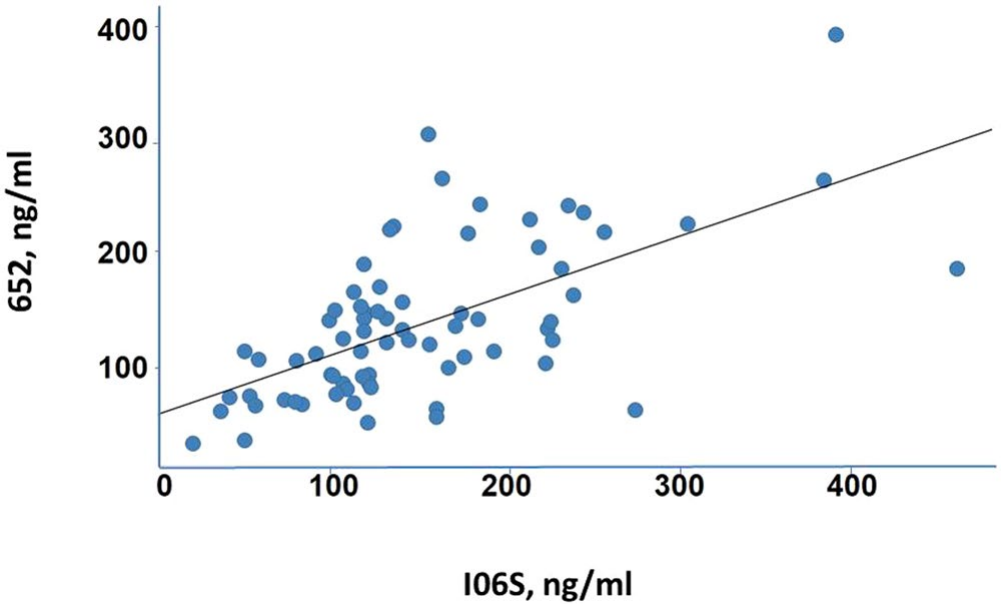
Comparison of BM652 levels in CSF from MPS I patients determined by our LC-MS method to the GAG level analysis using the Sensi-Pro assay (Arup). The continuous line, generated by linear regression (slope = 0.52, R^2^ = 0.39, n = 77), shows a positive correlation between the results obtained by the two methods (P < 0.0001).

Cerebral spinal fluid (CSF) provides a window to the condition of the central nervous system of MPS patients and a means for assessing treatment efficacy^22^. CSF levels of HS are elevated ~6-fold in MPS I patients^23^ and total GAGs are elevated 8- to 20-fold in MPS II patients^24^. So as a further validation of our method, we determined BM652 levels in 77 CSF samples drawn from: naïve MPS patients, patients at different intervals after HCT, and patients followed up to 2 years after ERT (IV or intrathecal administration). Sample volumes of 10 µl were sufficient for our assay. The full study on these patients will be presented elsewhere; here the purpose was to assess the sensitivity our LC-mass spec method and to compare the results to those obtained with the I0S6 Sensi pro method (Arup, Salt Lake City, Utah, also mentioned in^25,26^). In the I0S6 approach, non-reducing end disaccharides containing iduronic acid and sulfated glucose amine at position 6 are conjugated with a fluorescent label and quantified. We found that BM652 levels were linearly related to GAG levels in CSF as measured by I0S6 over a range exceeding 10–20-fold (Fig. 7). Given that numerous GAGs, including HS and our proposed structure for BM652, were tagged with the fluorescent label in the I0S6 method, whereas our method focused on BM652 alone, which is present at much lower levels than HS (Fig. 5), the comparison indicates that our method has considerably greater sensitivity for BM652 detection and quantification.

In conclusion, our analytical approach based on mass spectrometry of diseased tissues with minimal sample preparation revealed a battery of candidate biomarkers for MPS I. By probing two of these biomarkers, the method proved to be a sensitive, effective means for diagnosing MPS I and for following therapeutic interventions. Biopsied tissue was not required; very small volumes of plasma, urine or CSF were more than adequate. Discrimination of MPS type and early diagnosis is essential for tailoring the appropriate therapy. While GAG accumulation is characteristic of all forms of MPS, distinct biomarkers are expected because a different enzyme(s) is defective in each form. The approach outlined here for MPS I could be adapted to identify specific diagnostic biomarkers for other forms of MPS as well as for other lysosomal storage disorders. Because extensive sample preparation is unnecessary, testing could be done rapidly and at low cost.
