# Publisher Correction: Fast, sensitive method for trisaccharide biomarker detection in mucopolysaccharidosis type 1

**DOI:** 10.1038/s41598-018-23332-3

**Published:** 2018-03-19

**Authors:** Elina Makino, Helen Klodnitsky, John Leonard, James Lillie, Troy C. Lund, John Marshall, Jennifer Nietupski, Paul J. Orchard, Weston P. Miller, Clifford Phaneuf, Drew Tietz, Mariet L. Varban, Marissa Donovan, Alexey Belenki

**Affiliations:** 1 Drug Discovery, R&D, Sanofi, Waltham, MA 02451 USA; 2 Rare Disease, R&D, Sanofi, Framingham, MA 01701 USA; 3 Division of Pediatric Blood and Marrow Transplantation, University of Minnesota, Minneapolis, MN 55455 USA

Correction to: *Scientific Reports* 10.1038/s41598-018-22078-2, published online 27 February 2018

This Article contains an error in the order of the Figures. Figures 4 and 5 were published as Figures 5 and 4 respectively. The correct Figures appear below. The Figure legends are correct.

**Figure 4:**
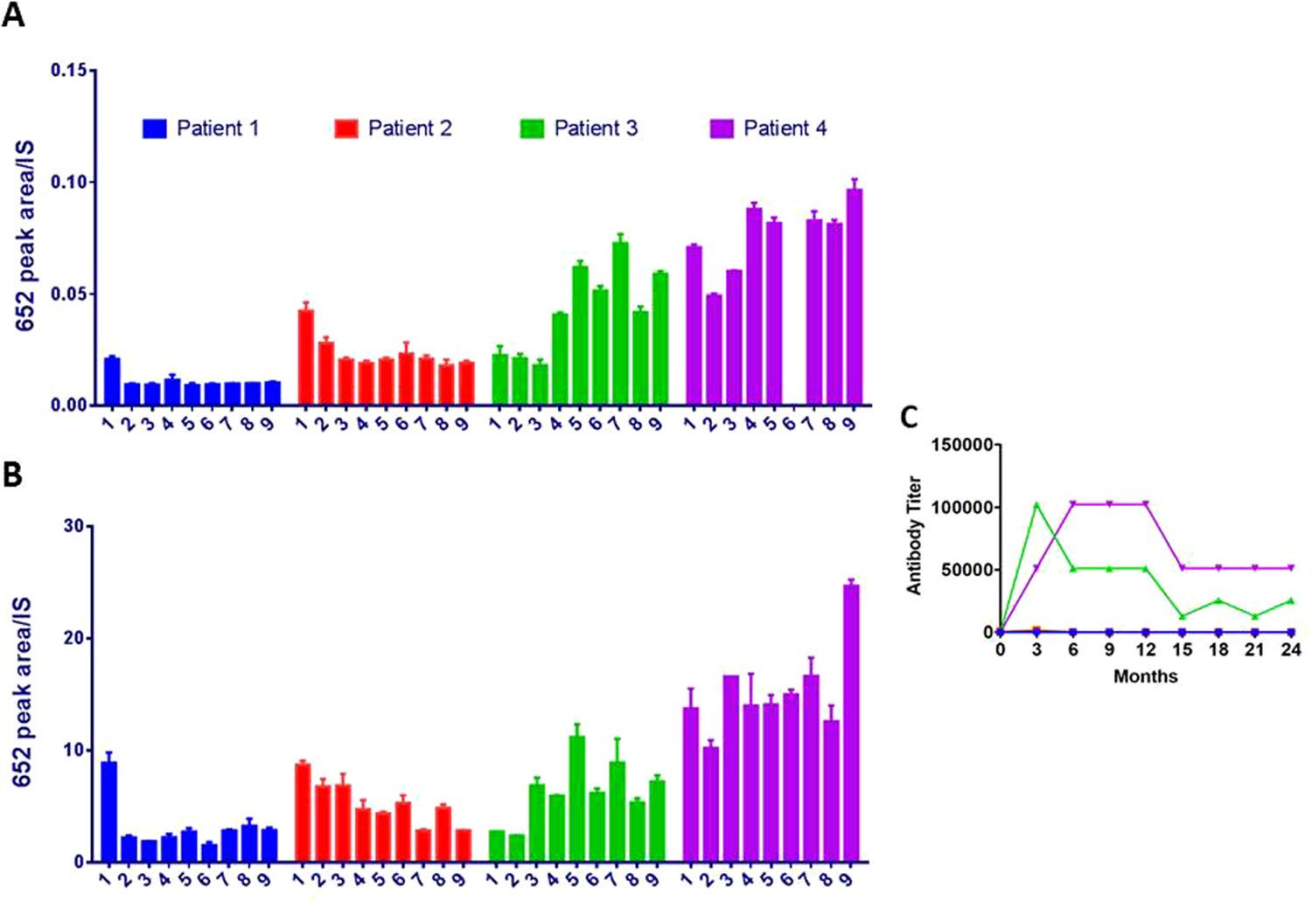
Validation of the methodology in (**A**) plasma and (**B**) urine of 4 human MPS I patients. All MPSI patients were treated >2 years after hematopoietic cell transplant (HCT) and were not receiving enzyme replacement therapy (ERT) in the interim. Patients had plasma and urine drawn at baseline (sample 1). Subsequently, patients went on ERT and had plasma and urine drawn at 3 month intervals for 24 months (samples 2 through 9). Plasma sample 6 of patient 4 is missing. n = 4 determinations were made for each sample point. (**C**) Surges in antibody titer for patients 3 and 4 as a function of time after initiation of ERT. Antibody was undetectable for patients 1 and 2 over the 24 month interval.

**Figure 5:**
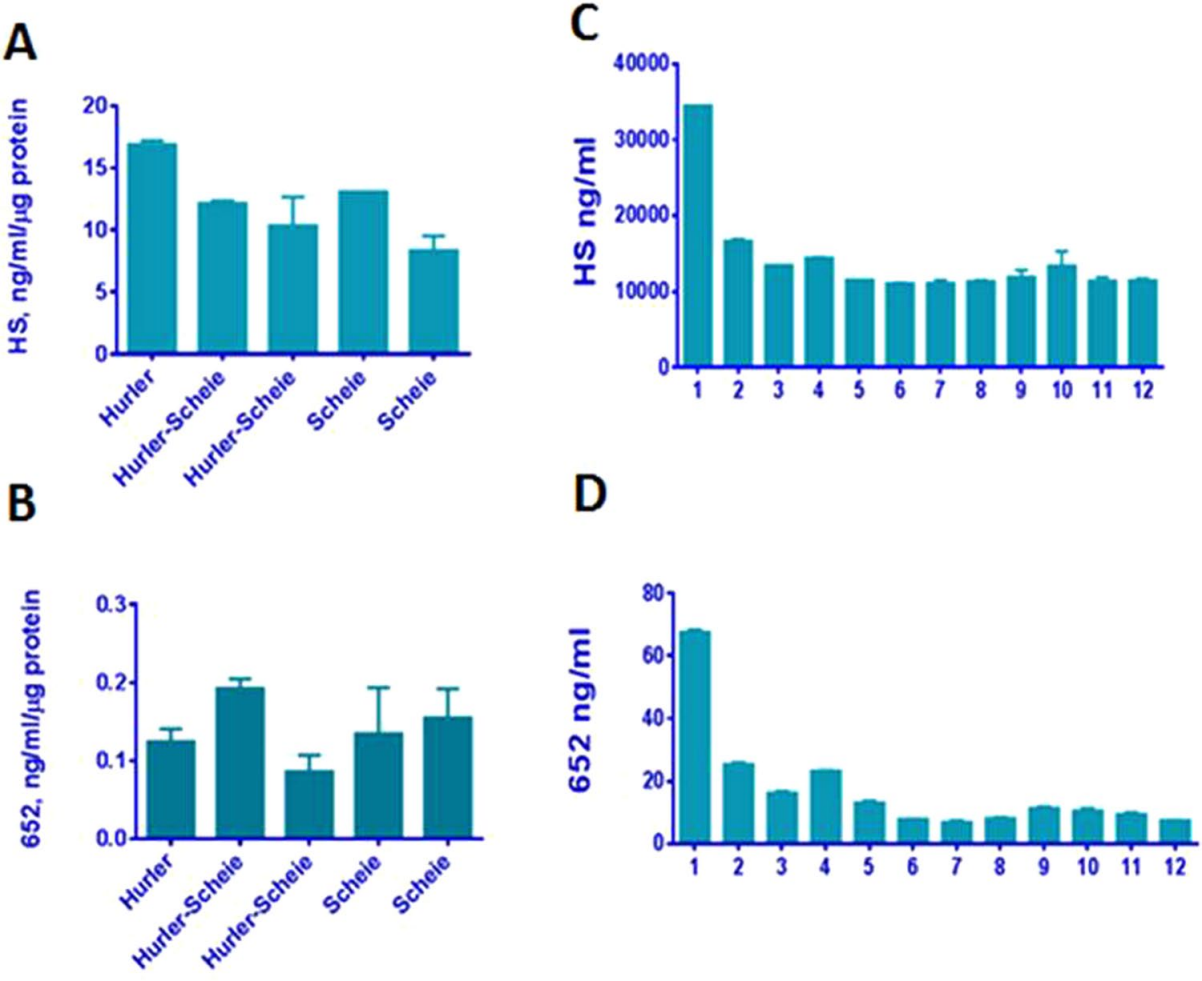
Quantification of heparan sulfate (**A**,**C**) and BM652 levels (**B**,**D**) in MPS I fibroblast cell lines (**A**,**B**) and in plasma of a human MPS I patient (**C**,**D**). Fibroblasts were derived from Hurler, Hurler-Scheie and Scheie patients. Samples 1–12 for MPS I patient (**C**) for HS and D for BM652) respectively: 1, enzyme naïve patient, 3 month prior to HCT; 2, 3 weeks pre HCT, patient on ERT; 3, 3 days prior HCT; 4, 7 days post HCT; 5, 21 days post HCT; 6, 41 days post HCT; 7, 49 days post HCT;8, 63 days post HCT; 9, 83 days post HCT; 10, 100 days post HCT; 11, 180 days post HCT, 12, 1 year post HCT.

